# Design and Preparation of Self-Oscillating Actuators Using Piezoelectric Ceramics with High Coupling Factors and Mechanical Quality Factors

**DOI:** 10.3390/mi13020158

**Published:** 2022-01-21

**Authors:** So-Won Kim, Hee-Chul Lee

**Affiliations:** Department of Advanced Materials Engineering, Korea Polytechnic University, Siheung 15073, Korea; swkim9193@kpu.ac.kr

**Keywords:** piezoelectricity, soft relaxor, piezoelectric actuators, coupling factors, mechanical quality factors

## Abstract

Piezoelectric material properties were optimized to develop materials for an ultrasonic vibrator targeting a high vibration efficiency. Herein, novel materials were developed using a composition represented by 0.08Pb(Ni_1/3_Nb_2/3_)O_3_-0.07Pb(Mn_1/3_Nb_2/3_)O_3_-0.85Pb(Zr_0.5_Ti_0.5_)O_3_ + 0.3 wt.% CuO + 0.3 wt.% Fe_2_O_3_ with 0.3 wt.% Sb_2_O_3_ doping. A ceramic shape with a thickness of 2 mm was optimized using finite element analysis software, and high values of coupling factors (0.54) and mechanical quality factors (1151) were obtained. This ceramic was used to fabricate a bio-beauty device (frequency = 1 MHz), and the manufactured ultrasonic vibrator indicated that the actuator oscillated with the maximum amplitude at a frequency of 1.06 MHz.

## 1. Introduction

Ultrasonic vibrators using piezoelectric ceramics are devices based on the reverse piezoelectric effect, which convert electrical energy into mechanical energy. When an AC voltage is applied, ultrasonic energy matching the signal frequency is emitted, which can be used as an ultrasonic vibrator [1]. Piezoelectric vibrators are compact and lightweight, owing to their simple structure, and have a wide frequency band; therefore, they have a wide variety of applications, depending on the size of the center frequency. Additionally, they can be used under different vibration modes, depending on the shape of the piezoelectric ceramics, such as cylindrical, disk, and curved types [2,3]. Among these, there is a high demand for disk-type piezoelectric ceramics because they have the advantage of generating high power in a low frequency range when combined with metals [4].

For a piezoelectric material to be applied as an ultrasonic vibrator, electrical energy needs to be appropriately converted into mechanical energy because the element operates using elastic vibrations, and it is essential to develop a material that can simultaneously satisfy the piezoelectric properties of the electromechanical coupling factor (k_t_) and the mechanical quality factor (Q_m_) for reducing the heat loss during high-power operations [5,6,7,8].

To develop a material that meets the application requirements, relaxor ferroelectrics, an ABO_3_ type complex perovskite compound, are dissolved in the basic composition of PZT piezoelectric ceramics, or the piezoelectric property values are modified via various means by using a small amount of additives; research on this was continuously being conducted [9,10,11,12,13,14].

This study aimed to develop an ultrasonic vibrator for a bio-beauty device (frequency = 1 MHz) that comprises a piezoelectric ceramic with a novel composition and metal processing material. The vibrator requires large amplitudes and high precision for the actuation of bio-beauty devices. First, to develop piezoelectric materials for ultrasonic vibrators, with different composition ratios of relaxor materials, piezoelectric properties were compared and analyzed with the simultaneous substitution of Pb(Mn_1/3_Nb_2/3_)O_3_(PMN), a hard relaxor, into Pb(Ni_1/3_Nb_2/3_)O_3_-Pb(Zr_0.5_Ti_0.5_)O_3_ (PNN-PZT). Moreover, we investigated the effect of doping with Sb_2_O_3_ to increase the mechanical quality factor, as a hardener. Then, using a finite element analysis software (ATILA FEA, Micromechatronics, State college, PA, USA), the measured values of the developed piezoelectric materials were examined, and a resonance mode analysis was performed according to the change in the thickness of the piezoelectric ceramics. Based on the analysis results, an actual ultrasonic vibrator was fabricated, and its performance was evaluated by analyzing the ultrasonic waveforms output during operation.

## 2. Materials and Methods

In this study, PbO, ZrO_2_, TiO_2_, Nb_2_O_5_, NiO, MnO_2_, CuO, and Fe_2_O_3_ were used as starting materials (high purity powders, ≥98%, Kojundo Chemical Laboratory); 0.3 wt.% CuO and 0.3 wt.% Fe_2_O_3_ were added as sintering aids to lower the sintering temperature in the basic composition, (0.15-x)Pb(Ni_1/3_Nb_2/3_)O_3_–xPb(Mn_1/3_Nb_2/3_)O_3_–0.85Pb(Zr_0.5_Ti_0.5_)O_3_ (PNN–PMN–PZT, x = 0.01–0.13). Raw material powder and zirconia balls were mixed together in an ethanol solution for 24 h to weigh the powder according to the composition ratio and disperse it evenly; thereafter, they were dried for 4 h or more at 120 °C. Then, the powder was calcinated at 850 °C for 4 h, and an excess doping content of Sb_2_O_3_ was added (0.1–0.7 wt.%) to the prepared powder and mixed for 24 h. An appropriate amount of 10 wt.% poly vinyl alcohol (PVA) aqueous solution was added to the powder as a binder, and a pressure of ~10 MPa was applied to form a disk (diameter = 20 mm). For the molded ceramic to have a denser structure, a water pressure of 130 MPa was applied via cold isostatic pressing (CIP) followed by sintering. Then, sintering was conducted at 500 °C for 1 h to remove the binder and then at 1000 °C for 2 h. The degree of densification was confirmed by observing the microstructure of the sintered specimen using scanning electron microscopy (SEM, ThermoFisher, Waltham, MA, USA); the crystallographic structure was analyzed using X-ray diffraction (XRD, BRUKER, Billerica, MA, USA) analysis. The average particle size was calculated via linear intercept analysis, determined by counting the number of grains intersecting the parallel lines on the SEM image and dividing the length of the parallel lines by the number of grains intersecting the lines.

To measure the electrical properties of the sintered specimen, both sides were polished, and then an Ag electrode was annealed at 650 °C for 30 min. Thereafter, for the polarization treatment, a 3 kV/mm electric field was applied for 30 min in a silicone oil bath at 120 °C, followed by aging for 24 h in air. For the polarized specimen, the piezoelectric constant (d_33_) was measured using a d_33_ meter (Model YE2730, HANTECH, Gunpo, Korea), and the electromechanical coupling factor (k_t_) and mechanical quality factor (Q_m_) were calculated according to the IEEE standard method using an impedance analyzer (Model 4990A, Keysight Technologies Inc., Santa Rosa, CA, USA). The Curie temperature was measured by the change of capacitance of the sample at 1 kHz in a chamber system (Poly K Technologies, State College, PA, USA) and by correlating the change in the dielectric constant with the temperature change from 20 to 500 °C using an LCR meter (E4980, Agilent, Santa Clara, CA, USA).

A piezoelectric ceramic made of the developed material optimized through the above experiment was attached to the center of a circular titanium workpiece; this workpiece remained in direct contact with skin in the beauty device via conductive epoxy. The manufactured ultrasonic vibrator element was assembled with a PCB circuit board that could auto-oscillate when power was supplied, and the frequency and output waveforms of the electrical signal generated from the ultrasonic vibrator were measured using an oscilloscope (TBS 2000 SERIES, Tektronix, Beaverton, WA, USA).

## 3. Results and Discussion

**Figure 1:**
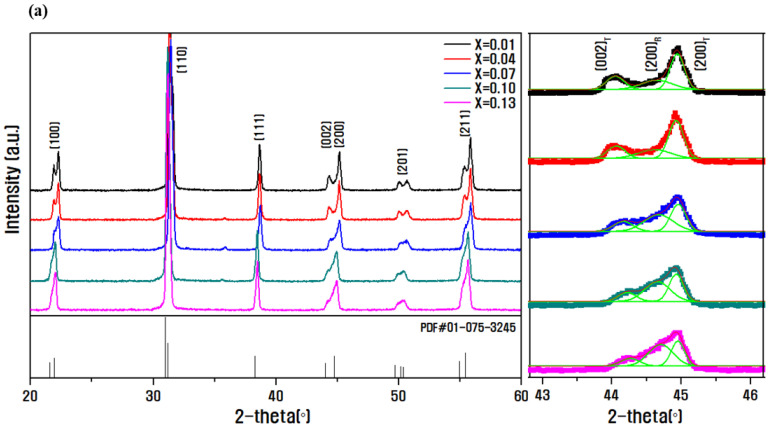

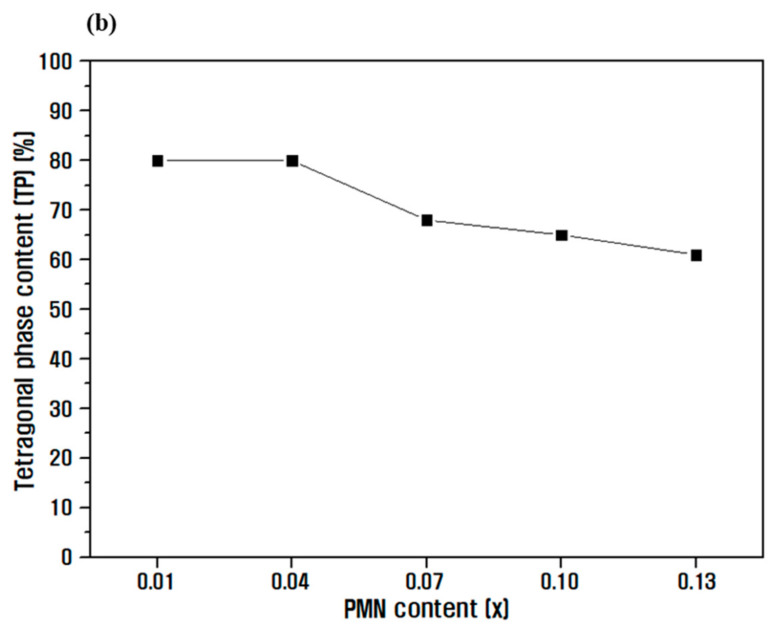
(**a**) X-ray diffraction pattern analysis results according to PMN molar ratio, and (**b**) tetragonal phase fraction results in the (0.15-x)Pb(Ni_1/3_Nb_2/3_)O_3_-xPb(Mn_1/3_Nb_2/3_)O_3_-0.85Pb(Zr_0.5_Ti_0.5_)O_3_ ceramic.

Figure 1 shows the XRD patterns of the sintered ceramic for different composition ratios of PNN and PMN. As shown in Figure 1a, a pure perovskite structure was formed without secondary phases under all composition ranges. A (200)_R_ rhombohedral diffraction line was formed with the increasing molar ratios of PMN, and phase change of the rhombohedral peak occurred due to the change in the crystal lattice when the diffraction pattern for 2-θ in the range of 43°–46° was enlarged. In Figure 1b, the relative intensity ratios of the tetragonal (002) and (200) peaks and rhombohedral (200) peak on the diffraction pattern are compared to calculate and show the tetragonal phase generation fraction of each specimen. The tetragonal phase content was calculated using the following formula:

(1) $$\mathrm{Tetragonal}\;\mathrm{phase}\;\mathrm{content}\;\left(\mathrm{TP}\%\right)=\frac{I_{\left(002\right)T}+I_{\left(200\right)T}}{I_{\left(002\right)T}+I_{\left(200\right)T}+I_{\left(200\right)R}}\times 100\%,$$

where I_(002)T_ and I_(200)T_ indicate the tetragonal (002) and (200) diffraction peak intensities, respectively, and I_(200)R_ denotes the rhombohedral (200) diffraction peak intensities [10]. The tetragonal phase fraction calculated decreased from 80% to 61% as the PMN molar ratio increased.

**Figure 2:**
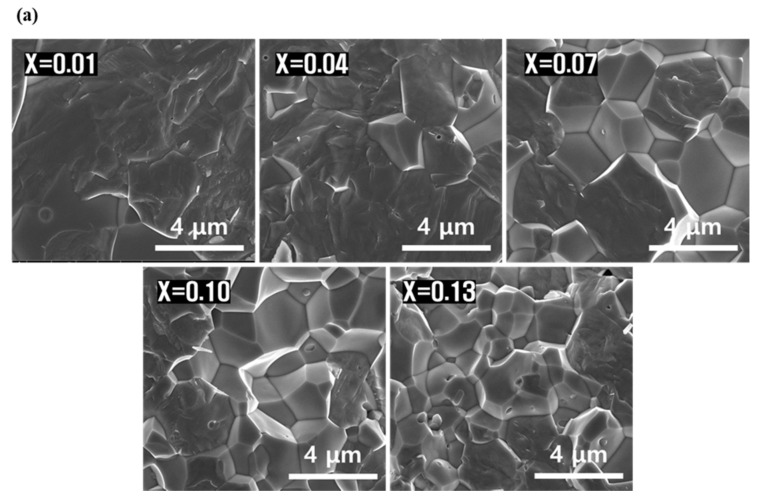

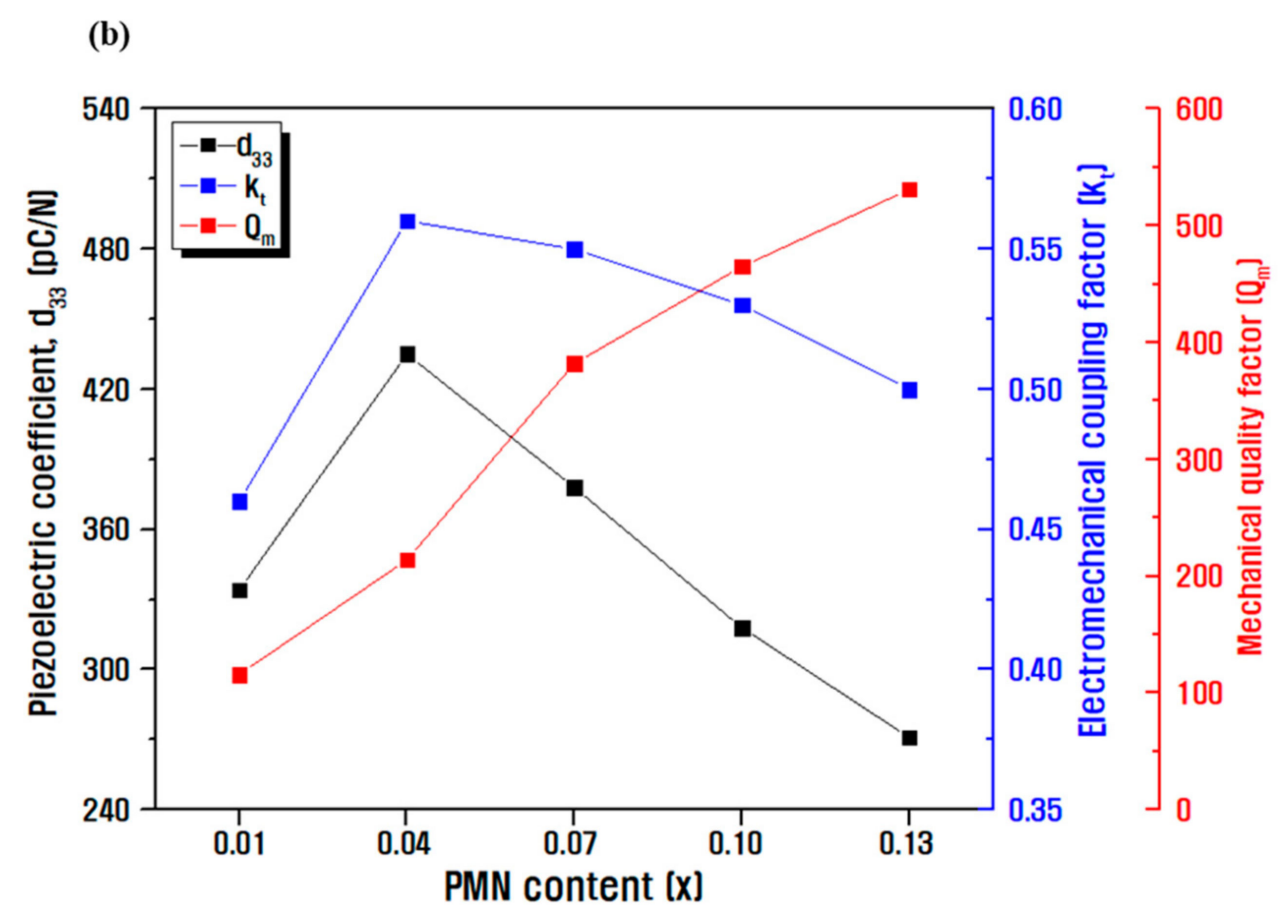
(**a**) FE-SEM images of fracture surfaces with different PMN molar ratios and (**b**) measurement results of electrical properties in (0.15-x)Pb(Ni_1/3_Nb_2/3_)O_3_-xPb(Mn_1/3_Nb_2/3_)O_3_-0.85Pb(Zr_0.5_Ti_0.5_)O_3_ ceramic.

Figure 2a shows SEM images of the microstructure of the fracture surface of the sintered PNN-PMN-PZT ceramics with different composition ratios. In certain portions of all compositions, grain boundaries were not observed. According to Li [15], this can be attributed to the addition of CuO and Fe_2_O_3_, which helps form a liquid phase at a low temperature. With PbO-CuO (680 °C) and PbO-Fe_2_O_3_ (730 °C), the bonding between the grain boundaries is strengthened, but the internal strength is weakened owing to internal defects, and the area of transgranular fractures increases [16]. Zheng et al. [17] reported that when rhombohedral phase stabilization is achieved, the particle size decreases owing to the improvement of internal stress. Although the grain size was not accurately measured owing to the transgranular fracture, the overall grain size tends to decrease as the PMN molar ratio increases.

Figure 2b shows the measurement results of electrical characteristics for different composition ratios. The piezoelectric coefficient and electromechanical coupling coefficient were maximum in the composition with x = 0.04; as the PMN molar ratio increased, these coefficients decreased in proportion to the particle size. A higher mechanical quality factor leads to changes to the domain motion, which is inversely proportional to the internal stress; therefore, as the PMN molar ratio increases, the particle size decreases, and the internal stress improves, making it difficult to move the domain. It is considered that the mechanical quality factor continues to increase [18,19,20,21].

A decrease in the tetragonal phase fraction is confirmed in Figure 1. In addition, to improve the value of the mechanical quality factor while maintaining the characteristic value at x = 0.07, along with excellent piezoelectric and electromechanical coefficients of 378 pC/N and 0.55, respectively, an experiment was conducted to add a doping material, Sb_2_O_3_, as a hardener.

**Figure 3:**
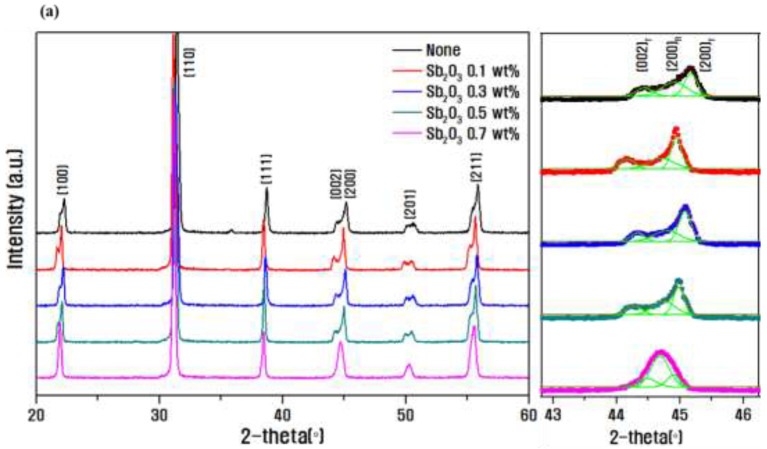

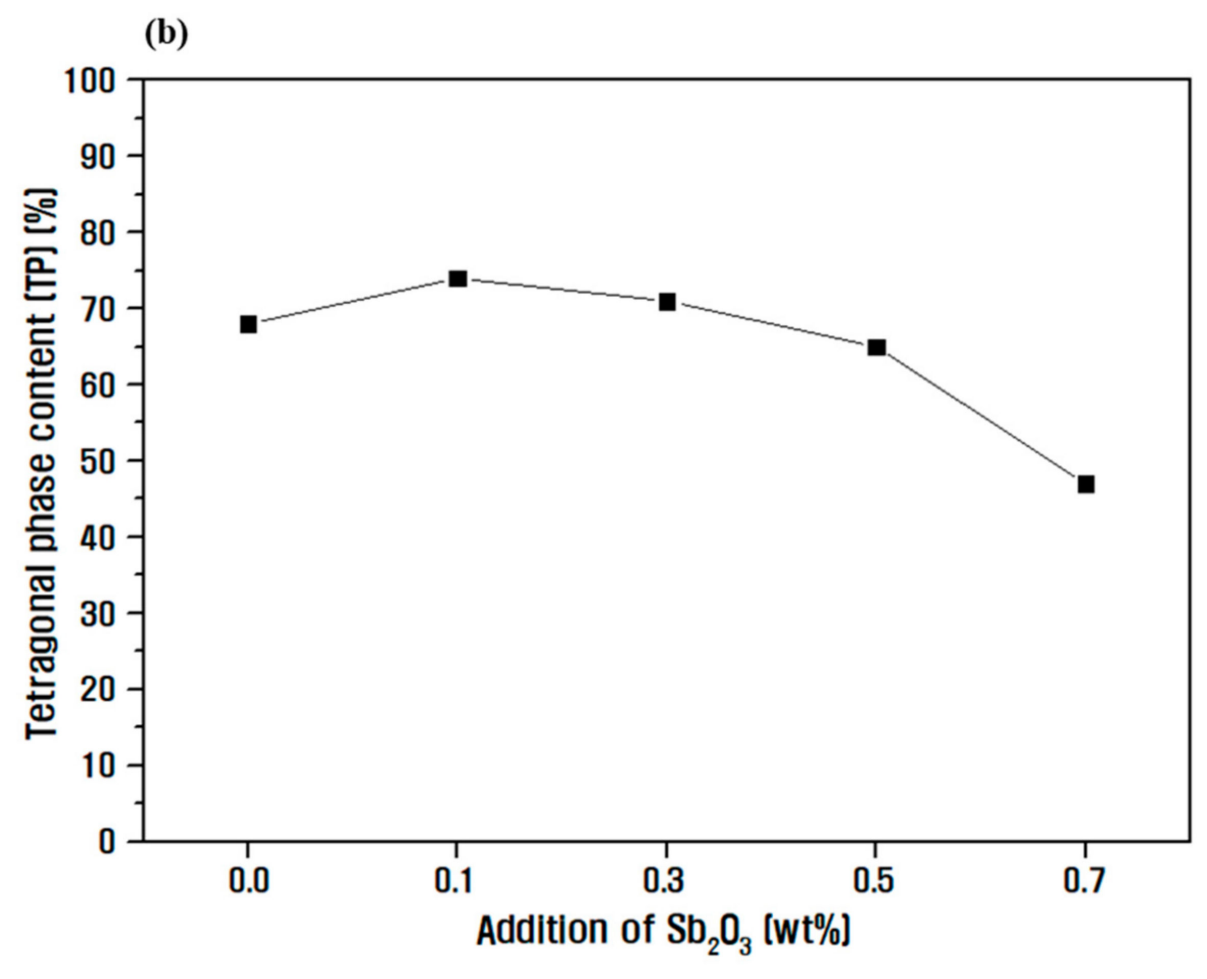
(**a**) Results of X-ray diffraction pattern analysis with different Sb_2_O_3_ doping amounts in 0.08Pb(Ni_1/3_Nb_2/3_)O_3_-0.07Pb(Mn_1/3_Nb_2/3_)O_3_-0.85Pb(Zr_0.5_Ti_0.5_)O_3_ ceramics and (**b**) tetragonal phase fraction results.

Figure 3 shows the XRD patterns with Sb_2_O_3_ as the dopant in 0.08PNN-0.07PMN-0.85PZT. In Figure 3a, when Sb_2_O_3_ was added, the Sb^3+^ (0.76 Å) ion had a similar ionic radius and occupied the high valence Zr^4+^ (0.72 Å) site, resulting in stabilizing the isotropic rhombohedral phase. With an increasing Sb_2_O_3_ dopant content, the (200)_R_ diffraction line between 43° and 46° was enhanced [22]. Figure 3b also quantitatively confirms that the tetragonal phase fraction gradually decreased due to the phase transition to the rhombohedral phase with increasing amounts of Sb_2_O_3_.

**Figure 4:**
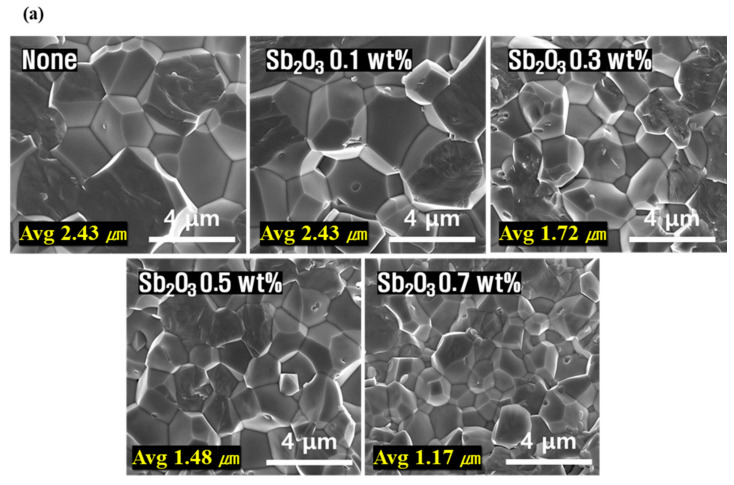

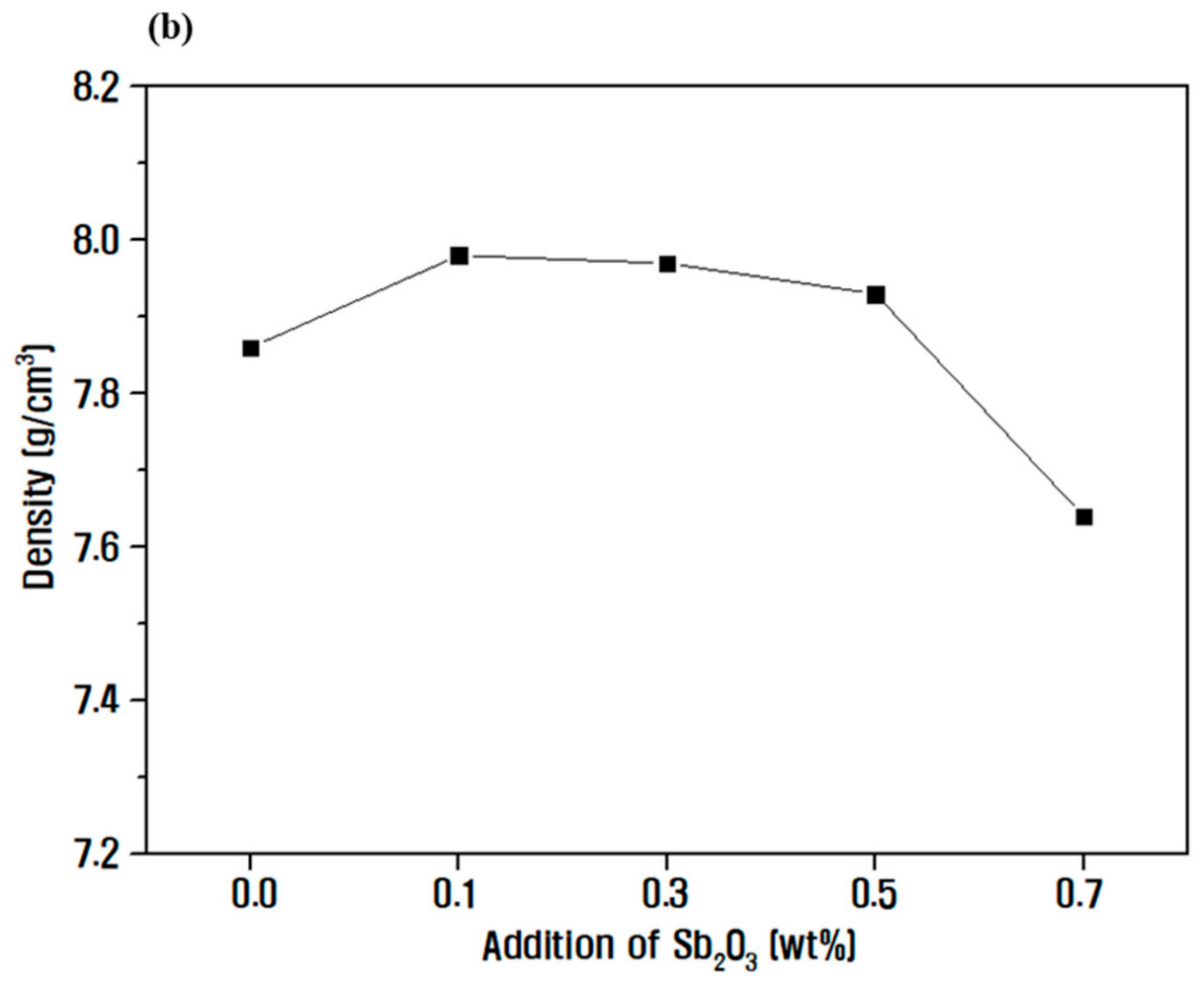
(Inset) FE-SEM images of fracture surfaces with different Sb_2_O_3_ doping amounts in 0.08Pb(Ni_1/3_Nb_2/3_)O_3_-0.07Pb(Mn_1/3_Nb_2/3_)O_3_-0.85Pb(Zr_0.5_Ti_0.5_)O_3_ ceramic; (**a**) average grain size and (**b**) density measurement results.

Figure 4 shows the SEM images and density of the microstructures of the fracture surfaces of PNN-PMN-PZT ceramics with different Sb_2_O_3_ doping amounts. Figure 4a shows a dense microstructure under all conditions; the average grain size decreased from 2.43 to 1.17 μm with increasing Sb_2_O_3_ doping amount [23,24]. Figure 3 confirms that the internal stress in the lattice improved with decreasing crystal size, and phase transition to the rhombohedral phase occurred. As seen in the SEM images, Figure 4b shows a dense structure without pores when Sb_2_O_3_ is added, resulting in an excellent value of ~98% relative density with a maximum value of 7.97 g/cm^3^. The high density can be related to the liquid phase formation due to the low melting point (656 °C) of Sb_2_O_3_. It was confirmed that the density also decreased with decreasing particle size.

**Figure 5:**
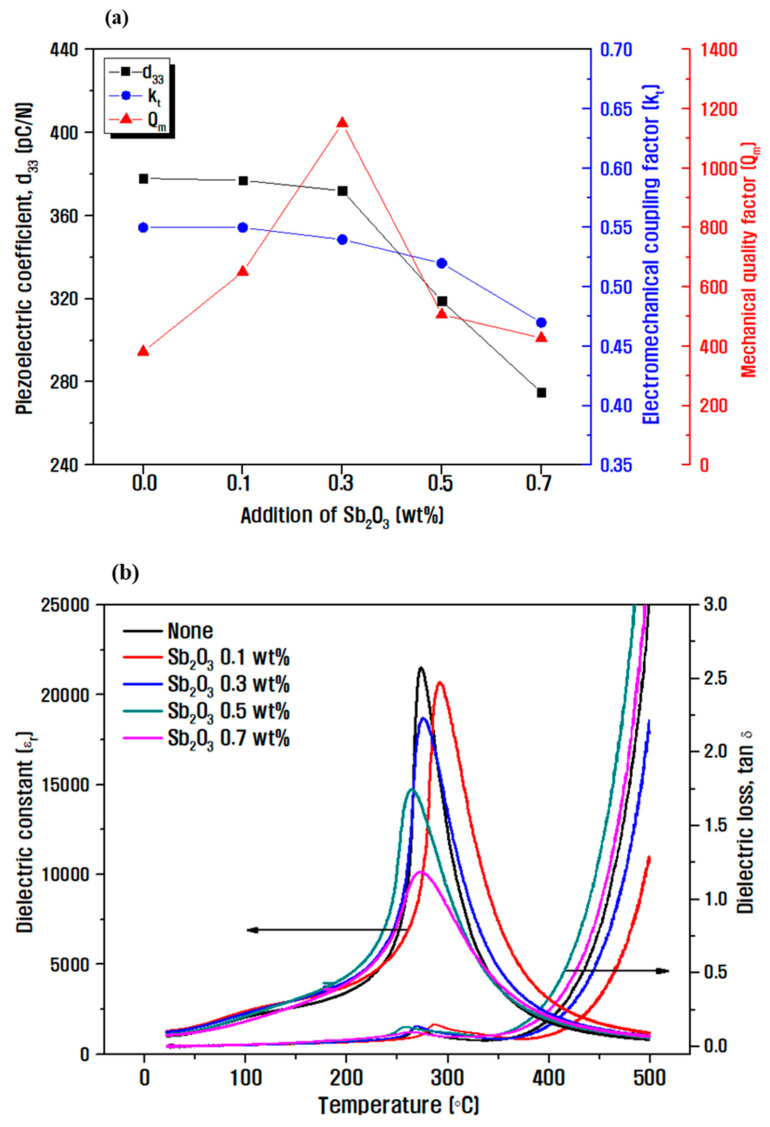
(**a**) Electrical characteristics of 0.08Pb(Ni_1/3_Nb_2/3_)O_3_-0.07Pb(Mn_1/3_Nb_2/3_)O_3_-0.85Pb(Zr_0.5_Ti_0.5_)O_3_ according to different Sb_2_O_3_ doping amounts, and (**b**) dielectric properties according to the temperature change at 1 kHz measurement.

Figure 5 shows the evaluated electrical characteristics for different Sb_2_O_3_ doping amounts. In Figure 5a, it is confirmed that the piezoelectric constant and electromechanical coupling coefficient decreased with decreasing polarization efficiency and average grain size due to the decrease in anisotropy with an increasing amount of Sb_2_O_3_. However, as Sb^3+^ was substituted at the B-site, space charges were formed owing to the occurrence of oxygen vacancies, and an internal electric field was generated in the ceramic particles, which suppressed the movement of the domains, increasing the mechanical quality factor. When the doping amount was >0.3 wt.%, the mechanical quality factor decreased, which implies a decrease with decreasing density [25]. Figure 5b confirms the Curie temperature at the point at which the dielectric constant is the highest, determined by measuring the dielectric properties according to the temperature change at 1 kHz. All the samples possessed a highly stable curve below the Curie temperature, indicating a good ferroelectric-stable characteristics. In the phase transition region, the movement of the interphase boundary induced an energy loss abruptly, leading to the loss peak around the Curie temperature. The Curie temperature was 275 °C at 0.3 wt.% Sb_2_O_3_, implying a maximum mechanical quality factor [26,27].

By adding 0.3 wt.% Sb_2_O_3_ doping material to the optimized composition of 0.08PNN-0.07PMN-0.85PZT, d_33_ = 372 pC/N, k_t_ = 0.54, the mechanical quality factor increased to 1151 without significant deterioration in properties compared to the basic composition, and thus, a piezoelectric material for ultrasonic vibrators was developed. These excellent piezoelectric properties show superior values compared to those of conventional commercially available materials; thus, the findings of this study are expected to provide insights for developing new compositions without the need for expensive rare elements.

Before manufacturing the ultrasonic transducer, simulation was performed using the piezoelectric analysis program (ATILA) to optimize the size of the piezoelectric body. The natural frequency of the piezoelectric ceramic was determined based on the piezoelectric constant and material dimensions, and each frequency region used different vibration modes. Typically, when an AC voltage is applied to piezoelectric ceramics, two resonance modes appear. First, the resonance occurring at the lowest frequency is a radial vibration mode, and at this time, the resonance and anti-resonance frequencies and resonance resistance values affect the electromechanical coupling coefficient and mechanical quality coefficient [28,29,30]. Second, the thickness vibration mode of piezoelectric ceramics occurring at high frequencies is the most important vibration mode for transmitting and receiving ultrasonic waves, and the center frequency of the vibrator strongly depends on the thickness of the piezoelectric ceramics [31]. Therefore, to analyze the resonance mode according to the change in thickness, the diameter (D) of the ceramic was fixed to 16 mm, which is the diameter after sintering, and the models were designed with four thicknesses (T), namely 0.8, 1.2, 1.6, and 2.0 mm. For the characteristic values such as d_33_, density, and dielectric constant of the piezoelectric ceramic, values measured from the previously optimized sample were input. To determine the frequency band where resonance occurred, modal analysis was conducted; it was confirmed that the largest resonance characteristic appeared with a coupling factor of 30% near 141 kHz. Based on the frequency values determined via modal analysis, the frequency range was determined, and then, harmonic analysis was performed to predict the resonance mode and impedance characteristics. 

**Figure 6:**
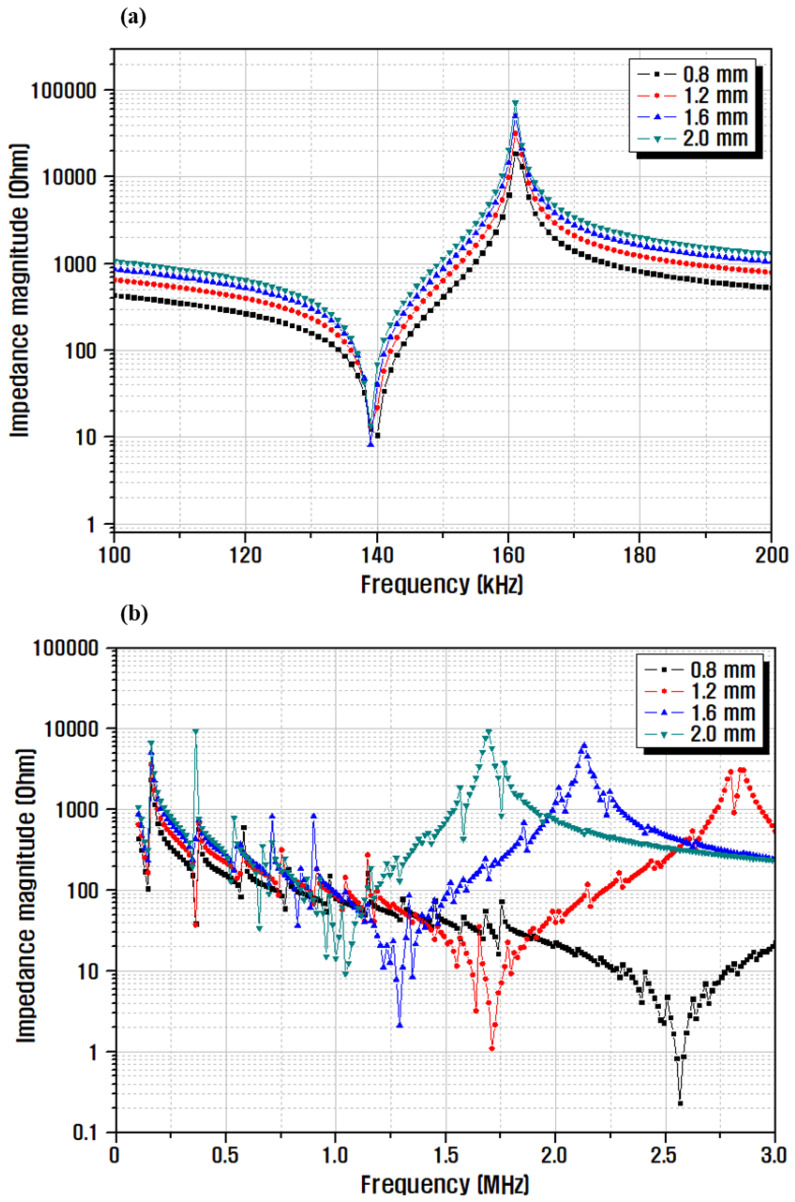
Simulation results of impedance characteristics for different thicknesses of piezoelectric ceramics: (**a**) radial, and (**b**) thickness vibration mode.

Figure 6 shows the results of the impedance characteristic analysis for different thicknesses of the piezoelectric ceramic via piezo simulation analysis and the results of the impedance analysis for operation of the ultrasonic vibrator element at different frequencies. In the radial vibration mode, the resonant frequency of 139 kHz and anti-resonant frequency of 161 kHz were the same irrespective of the different thickness of the ceramic at the same position. The result of analyzing the resonance mode occurred at a high frequency (Figure 6b). In the thickness vibration mode, for ceramic thicknesses of 0.8 and 2.0 mm, the center frequencies were 2.56 and 1.04 MHz, respectively, confirming the decrease in frequency [32,33,34].

**Figure 7:**
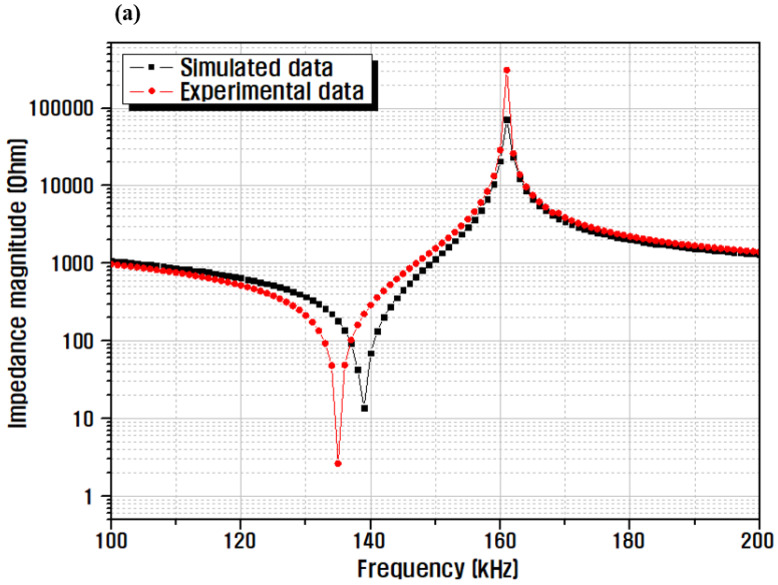

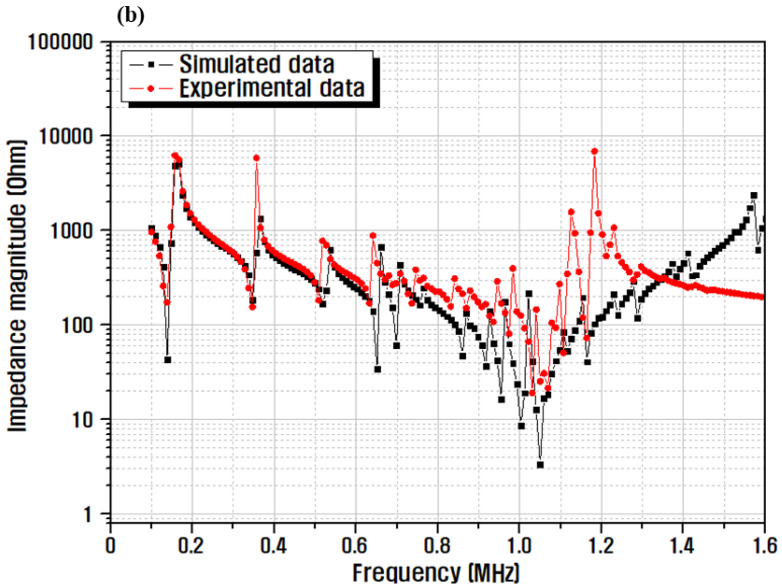
Simulation and impedance analysis results, according to the frequency of the fabricated sample: (**a**) radial, and (**b**) thickness vibration mode.

Figure 7 shows a comparison between the simulation and impedance analysis results for different frequencies using the fabricated sample. Beauty devices that are in contact with the skin typically use a frequency of 1 MHz; therefore, according to the simulation results, the piezoelectric ceramic was manufactured with a thickness of 2 mm, and the center frequency was 1 MHz. Comparing the impedance and simulation results in the radial vibration mode in Figure 7a, the resonant frequency of the actual sample was slightly lowered, from 139 to 135 kHz, and a low impedance of 2.6 Ω was obtained at that frequency. If the difference between the resonance frequencies of the radial vibration and the thickness direction vibration of the piezoelectric ceramic is >10 times, the radial vibration and thickness direction vibration do not affect each other. However, in the case of a ceramic with a thickness of 2 mm, as shown in Figure 7b, the center frequency in the thickness direction vibration mode is 1.06 MHz, and the difference in the resonance frequency in the radial vibration mode is small; therefore, the thickness direction vibration of the fabricated sample is mild. This also influences the directional vibration characteristics [35]. The center frequency in the vibration mode in the thickness direction of the actual sample exhibited a value almost identical to 1.04 MHz in the simulation analysis result; however, it can be seen that the difference in impedance value occurred when resonance occurs. This is attributed to the thickness deviation that occurs during grinding to 2 mm.

**Figure 8:**
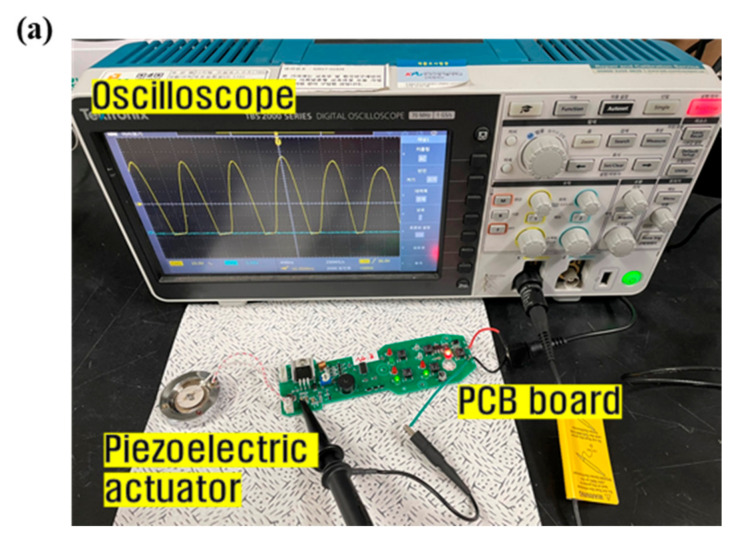

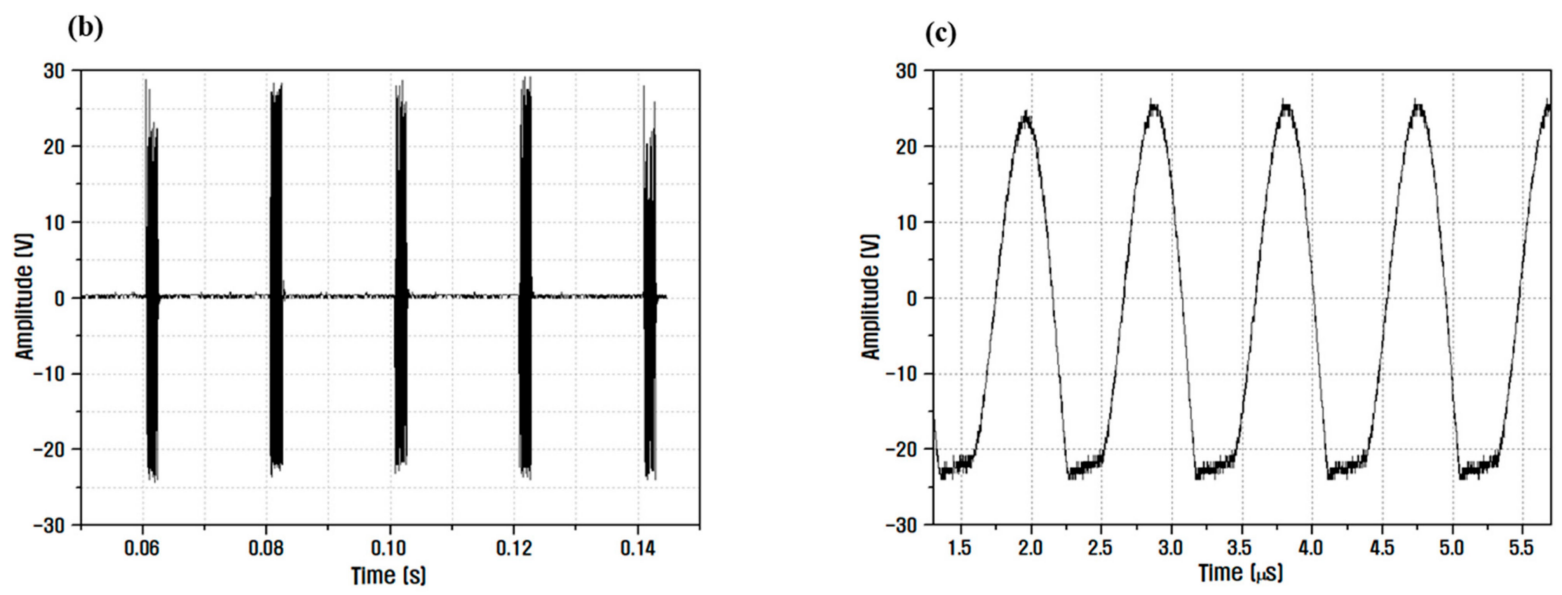
(**a**) Experimental apparatus for performance evaluation of ultrasonic vibrator, analysis results of (**b**) ultrasonic output waveform, and (**c**) detailed waveform.

The experimental apparatus for performance evaluation of the fabricated ultrasonic vibrator and the analysis results of the ultrasonic output waveform are presented in Figure 8. To measure the center frequency and waveform of the ultrasonic vibrator fabricated seen in Figure 8a, a self-oscillating PCB circuit that induces oscillation of the vibrator by generating a sinusoidal oscillation frequency and an ultrasonic vibrator were assembled. A voltage of 24 V was applied to the circuit, and the signal output from the oscillation of the ultrasonic vibrator at the center frequency was confirmed by connecting it to the oscilloscope equipment. By enlarging the detailed waveform of the modulated signal from the entire waveform in Figure 8b, the maximum amplitude was shown to oscillate at a frequency of 1.06 MHz, as shown in Figure 8c. The maximum output waveform yielded a value of 28 V. The simulation value was 1.04 MHz, but there was no significant difference at 1.06 MHz in the waveform measurement results of the fabricated sample.

## 4. Conclusions

In this study, to develop a material for an ultrasonic vibrator, piezoelectric material properties were optimized for different molar ratios of PNN and PMN, and varying amounts of Sb_2_O_3_ doping material were added for the optimized Pb(Ni_1/3_Nb_2/3_)O_3_-Pb(Mn_1/3_Nb_2/3_)O_3_-Pb(Zr_0.5_Ti_0.5_)O_3_ composition.

When 0.3 wt.% Sb_2_O_3_ dopant was added to the basic composition of 0.08PNN-0.07PMN-0.85PZT + 0.3 wt.% CuO + 0.3 wt.% Fe_2_O_3_, it was confirmed that the composition could be used in practical applications with a high-efficiency ultrasonic vibrator, as it exhibited excellent characteristic values of d_33_ = 372 pC/N, k_t_ = 0.54, Q_m_ = 1151, and T_c_ = 275 °C. As per the resonance mode analysis results for the different ceramic thicknesses, using the piezoelectric analysis tool, it was determined that the center frequency was 1.04 MHz when the ceramic diameter was 16 mm and thickness was 2 mm. An ultrasonic vibrator for a bio-beauty device was manufactured using the optimized ceramic material. Output waveform analysis of the manufactured ultrasonic vibrator revealed that the vibrator oscillated with maximum amplitude at a frequency of 1.06 MHz, which is almost identical to the designed value. Thus, by developing a material with a high electromechanical coupling coefficient, a high mechanical quality factor, and an optimized ceramic shape, an ultrasonic vibrator capable of stably generating ultrasonic waves at a target driving frequency was developed, and its applicability to various actuator devices was confirmed.

## Data Availability

The data presented in this study are contained within the article.
